# Expression profile and prognostic values of SMC family members in HCC

**DOI:** 10.1097/MD.0000000000031336

**Published:** 2022-10-21

**Authors:** Wei Yan, Dan-Dan Wang, He-Da Zhang, Jinny Huang, Jun-Chen Hou, Su-Jin Yang, Jian Zhang, Ling Lu, Qian Zhang

**Affiliations:** a Department of General Surgery, The First Affiliated Hospital of Nanjing Medical University, Nanjing, China; b Department of Surgery, the Johns Hopkins University, Baltimore, MD, USA; c Hepatobiliary Center of The First Affiliated Hospital, Nanjing Medical University & Research Unit of Liver Transplantation and Transplant Immunology, Chinese Academy of Medical Sciences, Nanjing, China; d The Affiliated Cancer Hospital (Jiangsu Cancer Hospital), Nanjing Medical, University, Nanjing, China; e State Key Laboratory of Reproductive Medicine, Nanjing Medical University, Nanjing, China.

**Keywords:** bioinformatics, HCC, SMC family, therapy

## Abstract

The structural maintenance of chromosome (SMC) gene family, including 6 proteins, is involved in a wide range of biological functions in different human cancers. Nevertheless, there is little research on the expression patterns, potential functions and prognostic value of SMC genes in hepatocellular carcinoma (HCC). Based on publicly available databases and integrative bioinformatics analysis, we tried to determine the value of SMC gene expression in predicting the risk of developing HCC.

**Methods:**

The expression and copy number variations data of SMC family members were obtained from TCGA (The Cancer Genome Atlas). We identified the prognostic values of SMC family members and their clinical features. GSEA (Gene Set Enrichment Analysis) was conducted to detect the mechanism underlying the involvement of SMC family members in liver cancer. We used Tumor Immune Estimation Resource database to explore the associations between TIICs (Tumor Immune Infiltrating Cells) and the SMC family members.

**Results:**

Our analysis proved that downregulation of SMC family members was common modification in HCC patients. In HCC, the expression of SMC1A, SMC2, SMC3, SMC4, SMC6 were upregulated. Upregulation of SMC2, SMC3, and SMC4, along with the clinical stage of HCC, were associated with a poor prognosis according to the results of univariate and multivariate Cox proportional hazards regression analysis. SMC2, SMC3, and SMC4 are also related to tumor purity and immune infiltration levels of HCC. The GSEA results proved that SMC family members take part in numerous biological processes underlying tumorigenesis.

**Conclusion:**

In this study, we comprehensively analyzed the expression of SMC family members in patients with HCC. This can provide insights for further investigation of the SMC members as potential therapeutic targets in HCC and suggest that the use of SMC inhibitor targeting SMC2, SMC3, and SMC4 can be a practical strategy for the therapy of HCC.

## 1. Introduction

Liver cancer is still a global health challenge, and the incidence rate is growing worldwide. The most common form of liver cancer is HCC (Hepatocellular carcinoma), HCC accounting for ~90% of liver cancer cases.^[1,2]^ In accordance with the World Health Organization’s statistics in 2018, HCC ranked sixth in incidence and fourth in mortality. There are about 840,000 new cases and over 780,000 deaths cases every year.^[3]^ In recent years, although some progress has been made through the comprehensive treatment based on surgical resection, the overall efficacy and prognosis of patients with HCC is still not ideal.^[4]^ Therefore, more development of prognostic biomarkers and therapeutic targets is required and may provide better prognosis and more effective precision medicine treatments for patients with HCC.

The structural maintenance of chromosome (SMC) is composed of 6 unique members (SMC1A, SMC2, SMC3, SMC4, SMC5, SMC6). SMC family members showed to be involved in DNA repair, sex-chromosome dosage compensation, genetic recombination, chromosome condensation and sister chromatid cohesion.^[5]^ Studies have shown that SMC family members have distinct and complex roles in different tumors. Upregulation of SMC family members have been frequently observed in different cancers and proposed to be potential therapy targets of diverse types of solid tumors.^[6–8]^ For example, it was reported that overexpressed phosphorylated SMC1A was significantly associated with poor prognosis in HCC,^[6]^ what’s more, hydrogen gas can inhibit lung cancer progression through targeting SMC3.^[7]^ It has been shown that condensin, a heterodimer composed of SMC4 and SMC2, is involved in chromatin condensation and gene regulation,^[9,10]^ high expression of SMC4 is an independent predictor of poor survival in patients with colorectal cancer, glioma and HCC.^[11–13]^ Studies also found that in the absence of the SMC5/6 complex, telomeres undergo progressive shortening in acute lymphoblastic leukemia cells, leading to cellular senescence, thus showed SMC5/6 assists in the maintenance of telomere length in acute lymphoblastic leukemia cancer cells, whereby these cells acquire unlimited replicative potential.^[14]^ In the meantime, some studies have also proved that downregulation of SMC family members resulted in enhanced oncogenic properties of cancer cells. For example, Previous reports showed that diminishing the expression of SMC2 gene could inhibit tumor growth in colorectal cancer and enhance apoptosis of neuroblastoma cells.^[15]^ However, the expression and prognostic role of SMC gene family in HCC patients remain abstruse. Studying the differential expression of SMC family members in HCC provides an opportunity to ameliorate our understanding of HCC development and make sense to the development of new therapeutic agents. In our study, ground on updated online databases and integrative bioinformatics analysis, we first comprehensively assessed the expression and prognosis of individual SMC family members.

## 2. Materials and Methods

### 2.1. Data collection

This study was approved by the First Affiliated Hospital of Nanjing Medical University. TCGA (The Cancer Genome Atlas) research network, conducted by the National Cancer Institute and National Human Genome Research Institute, has molecularly analyzed over 10,000 tumor patients and 20,000 primary cancer samples across 33 cancer types. Among which, 375 cases of HCC and the matching clinical parameters including gender, age, tumor (T) stage, node (N) stage, metastasis (M) stage were extracted (https://portal.gdc.cancer.gov/). Moreover, Genomic Data Commons Data Transfer Tool was used to download high-throughput sequencing (HTSeq) Fragments Per Kilobase of transcript per Million mapped reads (FPKM) data on the SMC family.

### 2.2. Analysis of SMC family members expression in HCC

Perl 5.26 software was used to obtain the SMC family mRNA expression levels from the HTSeq level 3 data. The differential expression of the SMC family between HCC tissues and normal tissues was analyzed utilizing the limma package in R 3.6.0 software and visualized via pheatmap package. The Human Protein Atlas database, which contains immunohistochemical and immunofluorescence expression data from different kinds of normal and cancerous tissues was used to provide us with immunohistochemical staining images of SMC family proteins in normal liver tissues and HCC tissues. Above all, we could compare different expression levels of SMC family between HCC tissues and normal tissues.

### 2.3. Survival analysis of SMC members in HCC

Two steps were used to explore the prognostic values of the SMC family members: by using univariate Cox proportional hazards regression analyses, the associations between SMC members, different clinical parameters and overall survival (OS) among HCC patients were investigated; Using multivariate Cox proportional hazards regression analysis, the independent prognostic values of the SMC members were then obtained by controlling the significant clinical parameters from step 1. All the analyses were accomplished by the use of the survival package in R 3.6.1 software. By these 2 different analyses, we could learn more about the survival rate of SMC members in HCC.

### 2.4. Immune cell environment analysis

The Tumor Immune Estimation Resource platform (https://cistrome.shinyapps.io/timer/), which is an integrated online tool for evaluating the abundance of tumor immune infiltrating cells (TIICs) through diverse cancer types, was utilized to detect the relationship between TIICs and SMC members. Totally 6 kinds of TIICs were explored including B-cells, CD4^ + ^T-cells, CD8^ + ^T-cells, dendritic cells, macrophages, and neutrophils. In addition, the purity of tumors was also accurately quantified in the database. The Tumor Immune Estimation Resource database could show more details of the immune cell environment.

### 2.5. Gene set enrichment analysis (GSEA)

GSEA (version 4.0.1; http://software.broadinstitute.org/gsea/index.jsp) was performed to explore the potential pathways of SMC members participating in the carcinogenesis of HCC. The annotated gene set file c2.cp.kegg.v7.0.symbols.gmt (from the Msig database) was used as the reference. A random combination number of 1000 permutations were performed and a false discovery rate <0.05 was used to identify the significantly enriched pathways. Using GSEA analysis, potential pathways related with SMC members in HCC could be further explored.

### 2.6. Software and statistical analysis

Perl 5.26 software was used to analyze The HTSeq FPKM mRNA data downloaded from the TCGA database. All analyses were performed using the R software (3.5.1, www.r-project.org) loaded with different packages, the limma package for the expression of SMCs, the corrplot package for the correlation between methylation and mRNA expression of SMCs, the survival package for the analysis of prognostic values, the ggplot package for the multivariate Cox proportional hazards regression analysis. *P* < .05 was considered to be statistically different.

## 3. Results

### 3.1. mRNA expression of SMC members in HCC

**Figure 1.**
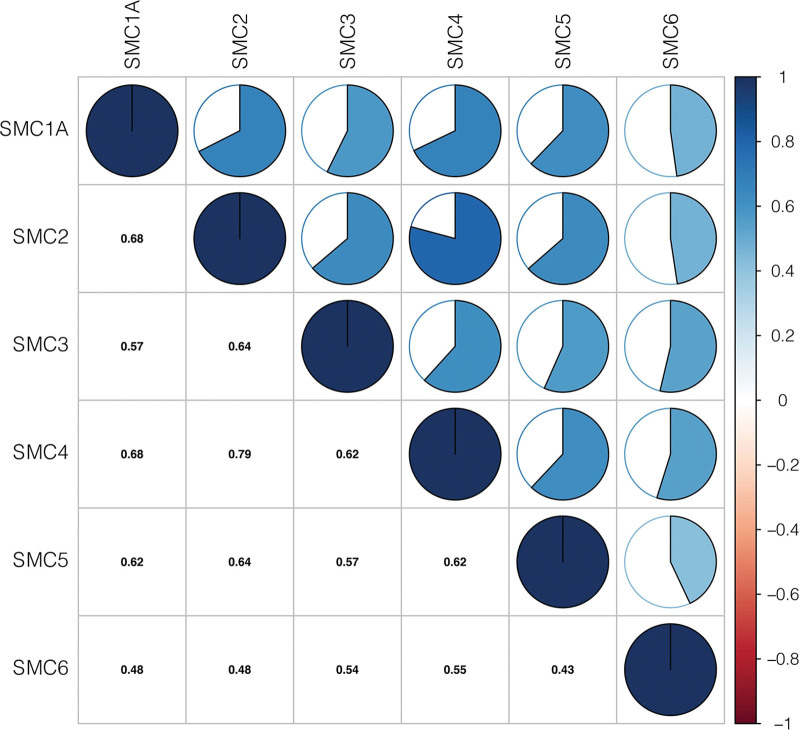
Associations between SMC family members. SMC = structural maintenance of chromosome.

To explore the mRNA expression level of SMC family members (SMC1A, SMC2, SMC3, SMC4, SMC5, SMC6) in HCC, Perl software was used to obtain the expression data of SMCs in 375 HCC cases and 50 normal tissues derived from TCGA. Interestingly, as shown in Figure 1, there is a significant correlation between SMC family members.

**Figure 2.**
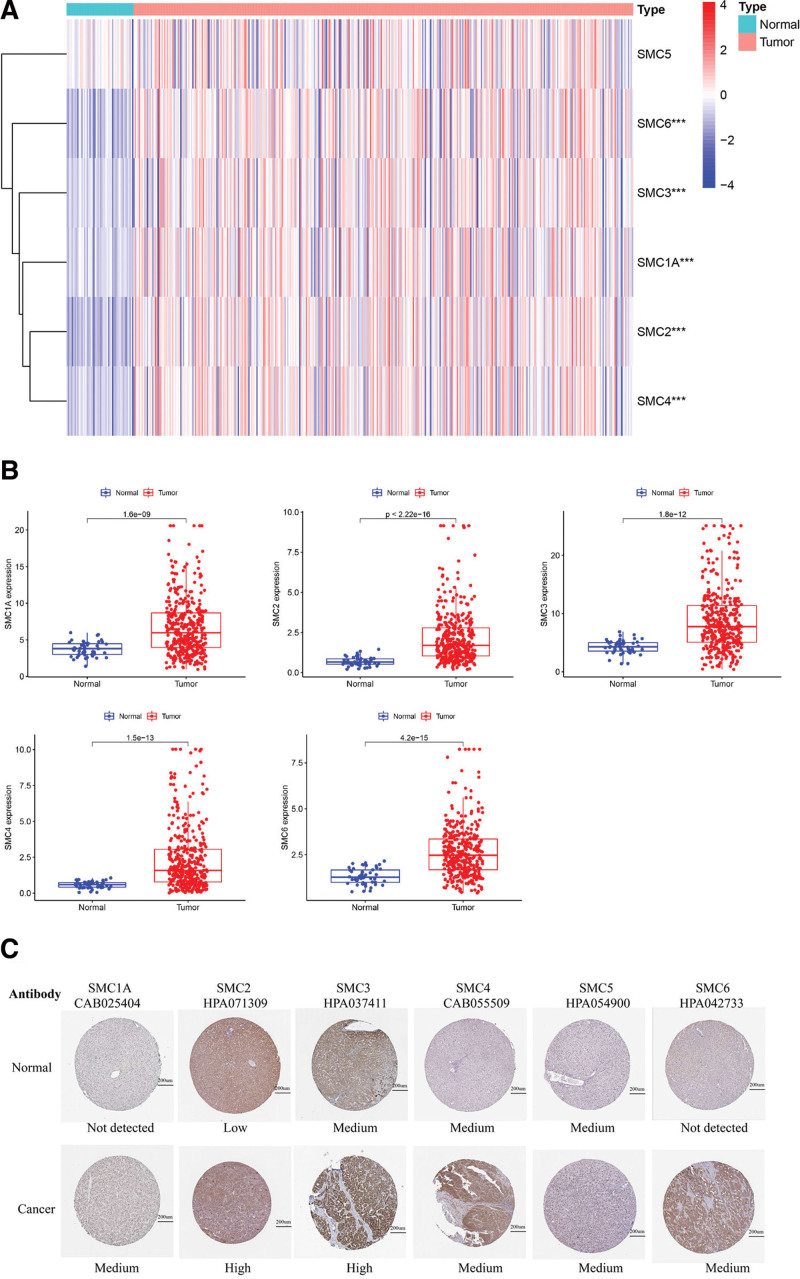
Expression profile of SMC members in HCC represented by a heatmap (A), histograms (B), and immunohistochemistry (C). HCC = hepatocellular carcinoma, SMC = structural maintenance of chromosome.

As shown in Figure 2A, limma package was used to show the differentially expressed SMCs and pheatmap package was used to visualize the results. As shown in Figure 2B, SMC1A, SMC2, SMC3, SMC4, and SMC6 were significantly upregulated in HCC tissues, whereas no difference was found in SMC5 expression between HCC and normal tissues. Moreover, the Human Protein Atlas database was used to explore the protein levels of SMCs in HCC. Next, in order to study the Protein levels of the SMC gene family in liver cancer tissues, we demonstrated the differences in the expression of SMC family proteins in HCC and normal liver tissues through the Human Protein Atlas database. As shown in Figure 2C, the expressions of SMC1A, SMC2, SMC3, SMC4, and SMC6 in HCC tissues were higher than those in normal liver tissues, while there was no significant difference between SMC5 in liver cancer tissues and normal tissues.

### 3.2. Prognostic values of SMC family members in HCC

**Figure 3.**
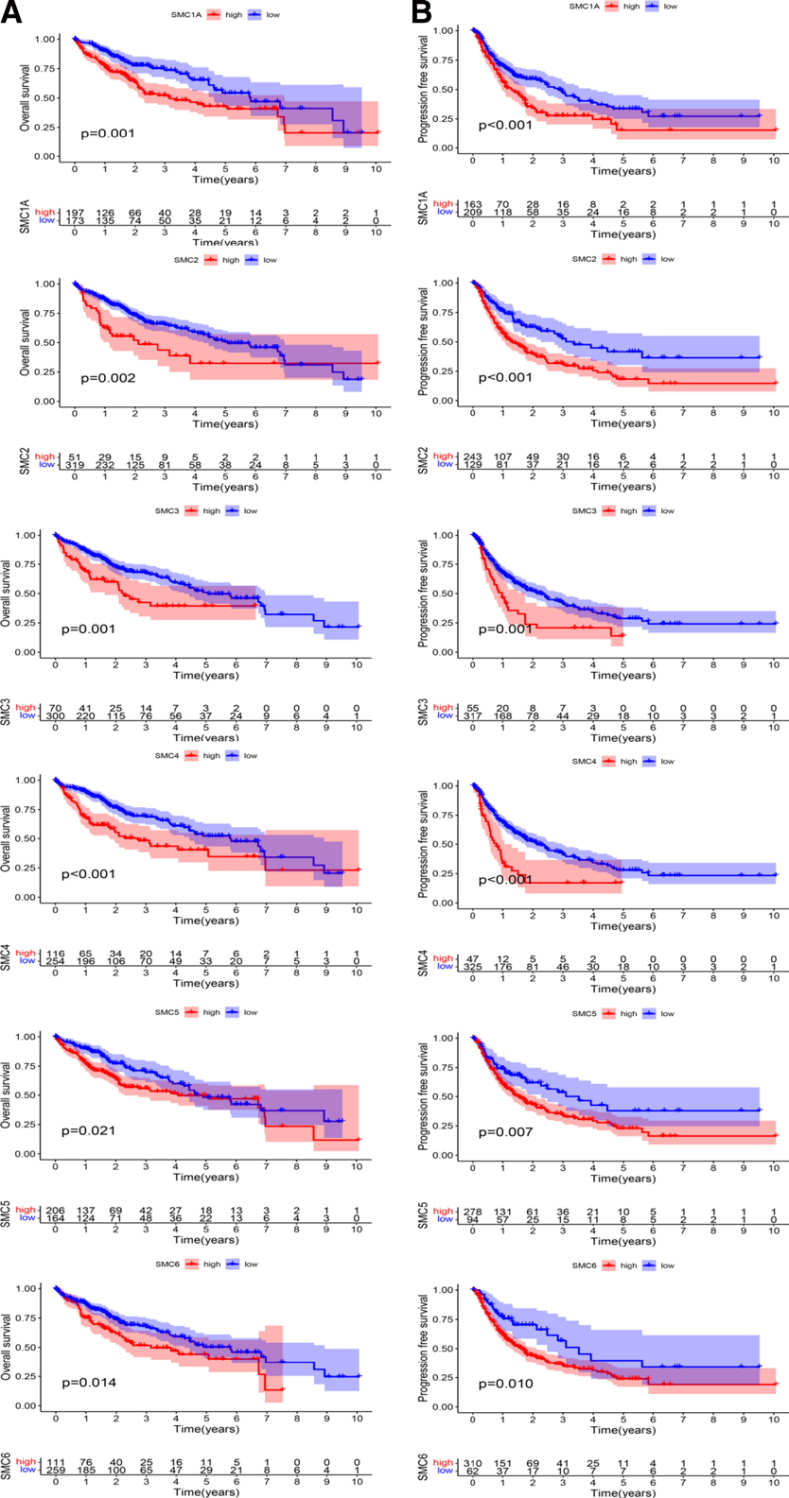
The prognostic value of mRNA Level of SMC factors in HCC patients. (A) The prognostic value of mRNA level of SMC factors in overall survival (OS) of HCC patients, (B) The prognostic value of mRNA level of SMC factors in progression-free survival (PFS) of HCC patients. HCC = hepatocellular carcinoma, SMC = structural maintenance of chromosome.

**Figure 4.**
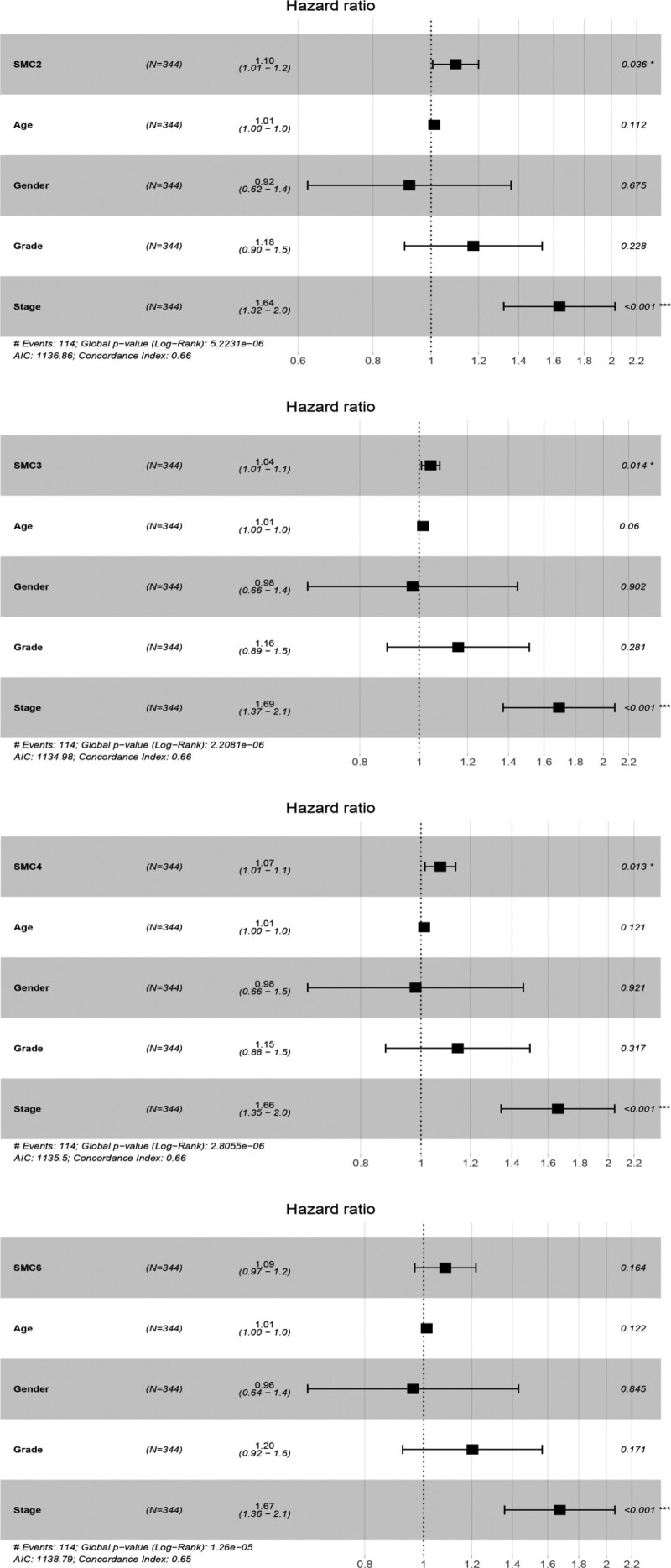
Forest plots of the results of multivariate Cox regression analyses of significant prognostic factors: SMC2 (A), SMC3 (B), SMC4 (C), and SMC6 (D). *P < .05; **P < .01. SMC = structural maintenance of chromosome.

Next, the prognostic significance of individual SMC members for HCC patients were further assessed. It was found that 4 SMC members (SMC2, SMC3, SMC4, SMC6) and 1 clinical feature (stage) were closely associated with poor outcome of HCC patients (hazard ratio [HR] for SMC2: 1.137 [1.051–1.230]; HR for SMC3: 1.041 [1.009–1.075]; HR for SMC4: 1.094 [1.038–1.152]; SMC6: 1.119 [1.001–1.250]); HR for stage: 1.679 (1.368–2.061) (Table 1). In order to further explore the relationship between the expression level of SMC members and the OS and progression-free survival (PFS) of HCC patients, we analyzed the expression profile and clinical data of HCC patients from TCGA database. The results showed that elevated expression of SMC2, SMC3, SMC4, and SMC6 in HCC patients were negatively correlated with patients’ OS, while higher expression levels were associated with shorter OS (*P* values of .002, .001, <.002, and .014), as shown in Figure 3A. Meanwhile, high expression of SMC2, SMC3, SMC4, and SMC6 was also negatively correlated with PFS, with higher expression levels associated with shorter PFS (*P* values of <.001, .001, <.001, and .01), as shown in Figure 3B. Next, multivariate Cox proportional hazards regression analysis was used to evaluate the independent prognostic values of SMC2, SMC3, SMC4 to control the influence of clinical features on the prognosis. The results, which are exhibited in forest plots (Fig. 4), confirmed that the expression of SMC2, SMC3, and SMC4 and a clinical parameter (stage) were independent biomarkers for the prognosis of HCC patients.

**Table 1:** Univariate cox proportional hazards regression analyses of SMC members and clinical features in HCC (bold means *P* < .05).

Parameter	Univariate analysis			
	HR	HR.95L	HR.95H	*P* value
SMC1A expression	1.038	0.997	1.080	.068
SMC2 expression	1.138	1.052	1.231	**.001**
SMC3 expression	1.042	1.009	1.076	**.012**
SMC4 expression	1.094	1.039	1.152	**.001**
SMC5 expression	1.063	0.975	1.160	.168
SMC6 expression	1.119	1.002	1.250	**.046**
Age	1.010	0.996	1.025	.174
Gender	0.776	0.531	1.132	.188
Grade	1.133	0.881	1.457	.330
Stage	1.680	1.369	2.062	**6.973E-07**

HCC = hepatocellular carcinoma, HR = hazard ratio, SMC = structural maintenance of chromosome.

### 3.3. Correlations between SMC members and TIICs in HCC

**Figure 5.**
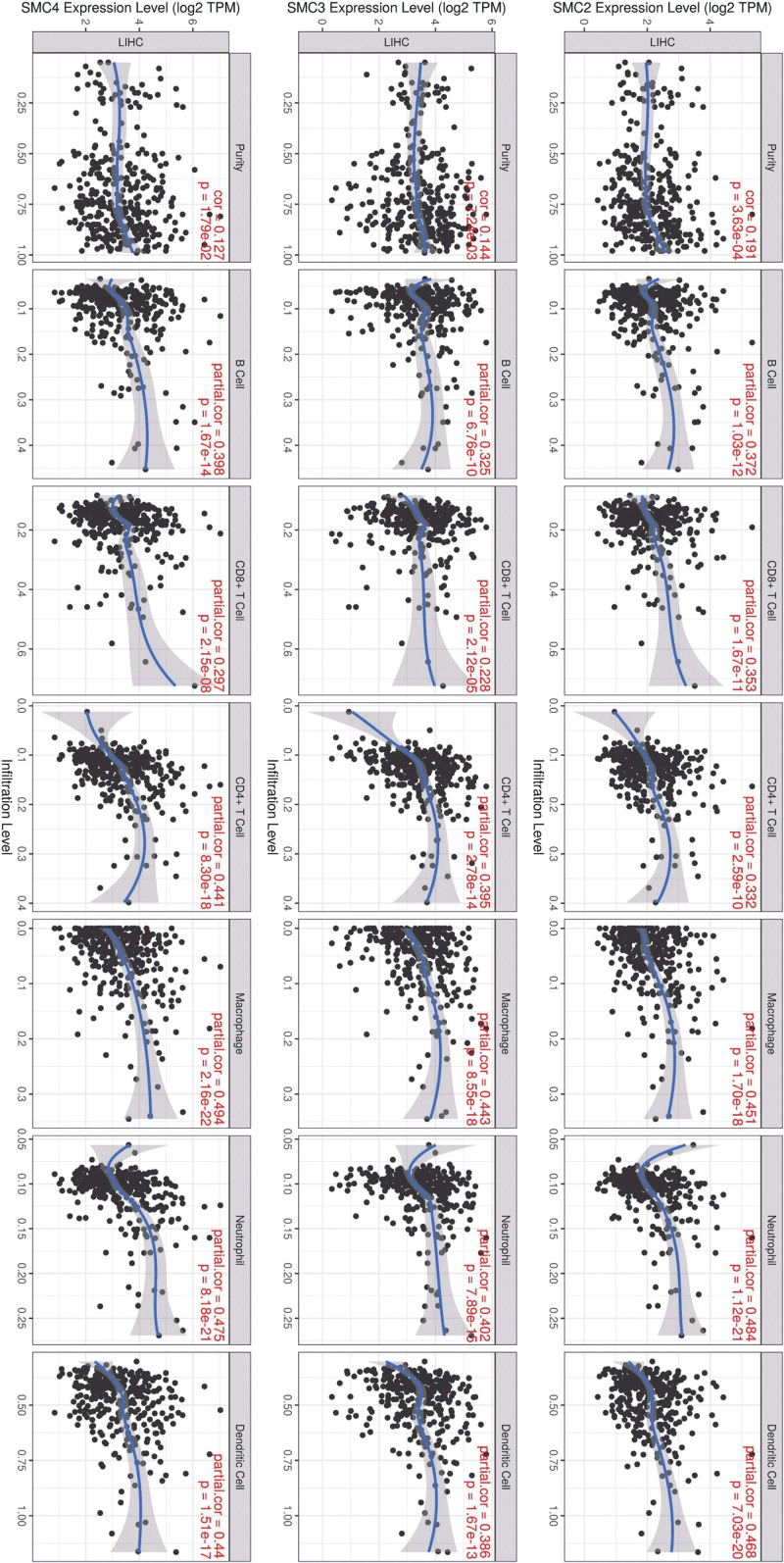
Correlations between tumor infiltrating immune cells (TIICs; B cells, CD4 + T cells, CD8 + T cells, neutrophils, macrophages, and dendritic cells) and SMC members (including SMC2, SMC3 and SMC4) in HCC. Tumor purity is shown in the panels on the left. HCC = hepatocellular carcinoma, SMC = structural maintenance of chromosome.

TIICs, an important part of the complex tumor microenvironment, have been reported to be associated with the progression of many cancers. Hence, it would be meaningful to explore the association between immune infiltration and SMC members. We calculated the coefficient of SMCs and the level of immune infiltration in HCC patients to determine whether they were related. The first column in Figure 5 showed significant correlations between SMCs expression and tumor purity. In addition, SMC expression was notably correlated with the infiltration levels of most immune cell types in HCC. Specifically, the level of SMC2 expression positively correlated with the infiltration levels of B cells (*R* = 0.372, *P* = 1.03e-12), CD8 + T cells (*R* = 0.353, *P* = 1.67e-11), CD4 + T cells (*R* = 0.332, *P* = 2.59e-10), macrophages (*R* = 0.451, *P* = 1.70e-18), neutrophils (*R* = –0.484, *P* = 1.12e-12), and dendritic cells (*R* = 0.468, *P* = 7.03e-20). The same thing with SMC3 and SMC4. These results indicated that 3 members of SMC gene family were related with tumor purity and immune infiltration levels in HCC.

### 3.4. Mechanism underlying the prognostic role of SMC members in HCC

**Figure 6.**
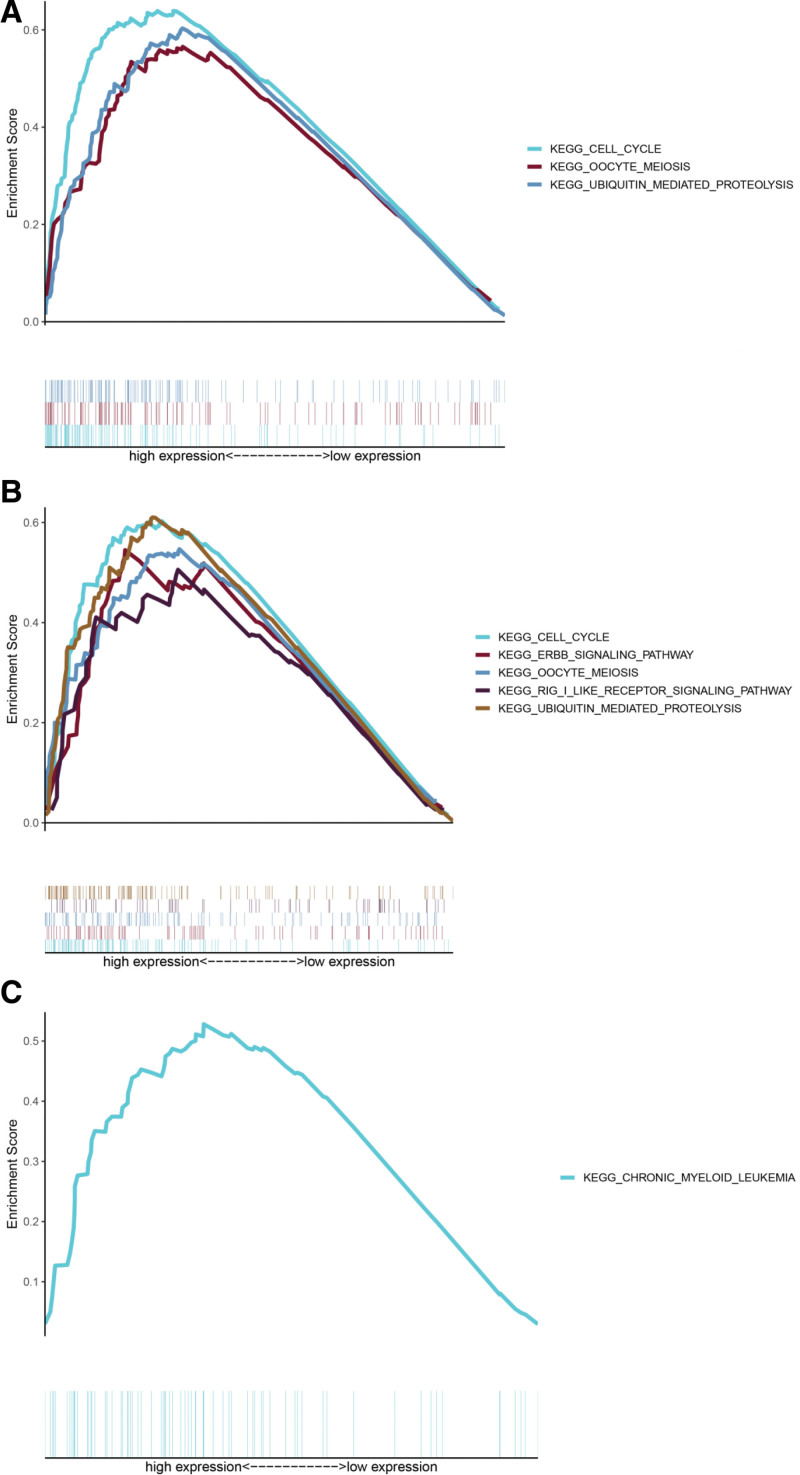
Cancer-related Kyoto encyclopedia of genes and genomes (KEGG) pathways associated with SMC2 (A), SMC3 (B), and SMC4 (C) based on a gene set enrichment analysis (GSEA). SMC = structural maintenance of chromosome.

We conducted a GSEA of differentially expressed SMC members with statistical prognostic value to detect the feasible biological mechanism by which differential expression of SMC2, SMC3, and SMC4 influences the carcinogenesis of HCC. The GSEA figured that high expression of SMC2 was related to “cell cycle,” “oocyte meiosis,” and “ubiquitin mediated proteolysis” (Fig. 6A), high expression of SMC3 was related to “cell cycle,” “ERBB signaling pathway,” “oocyte meiosis,” and “ubiquitin mediated proteolysis” (Fig. 6B), high expression of SMC4 was related to “chronic myeloid leukemia” (Fig. 6C).

## 4. Discussion

Bioinformatics is a new subject that refers to the application of informatics to the field of biology and is widely used to explore the mechanisms of carcinogenesis.^[16–18]^ Overexpression of SMC factors has been reported in many tumors. Increasing evidence has shown that the protein products of SMC genes not only act as transcriptional factors promoting carcinogenesis but also serve as tumor-suppressor factors, based on their aberrant expression patterns in certain organs.^[7,15,19,20]^ The availability of public genomic data provided us the opportunity to explore the expression profiles of families of genes in human cancers and their clinical practice value. This study evaluates the distinct expression and methylation profile, biological processes, and prognostic values of 6 SMC members expressed in HCC.

From the data, we found that the SMC family members likely contributed to carcinogenic effects in the development of HCC. Compared with normal tissues, all SMC genes with the exception of SMC5 were highly expressed in HCC cells. Moreover, the overexpression of SMC1A, SMC2, SMC3, SMC4, and SMC6 were related to the PFS of HCC patients. In this study, univariate Cox proportional hazards regression analyses were performed to analyze the prognostic values of SMC members in HCC. In fact, 4 SMC members were significantly associated with poor clinical outcomes in HCC (SMC2, SMC3, SMC4, SMC6). In addition, multivariate Cox proportional hazards regression analyses affirmed the prognostic value of SMC2, SMC3, SMC4 in the prediction of poor outcome in HCC patients.

The SMC 1 alpha (SMC1A) gene is located in Xp11.22-p11.21 and consists of 25 exons and 24 introns.^[21]^ It is a member of SMC family that serves critical roles in organizing and stabilizing chromosomal segregation during mitosis, and is considered to be a component of the signaling network involved in the maintenance of genome stability.^[22]^ SMC1A is known for its role in cell division, DNA repair and activation of cell cycle checkpoints.^[23,24]^ Previous studies reported that upregulated SMC1A may be associated with glioblastoma, lung cancer and colon cancer progression,^[25–27]^ but there is little information on the expression and prognosis of SMC1A in HCC. In this study, we analyzed TCGA datasets and found that SMC1A was more highly expressed in HCC tissue compared to normal tissue, and that increased expression of SMC1A is significantly associated with poor PFS.

SMC2 forms part of the condensin complex that has a central role in many aspects of chromosome biology, including the segregation of sister chromatids and compaction of chromosomes during cell division, as well as the regulation of gene expression during the interphase.^[9,28–30]^ SMC2 has been found to be over-expressed in a significant number of patients with colorectal cancer, gastric cancer, lymphoma and some types of neuroblastoma,^[31,32]^ and has been suggested as a risk biomarker in pancreatic cancers.^[33]^ SMC3 is involved in DNA damage repair^[23]^ and microtubule-based intracellular transport.^[34,35]^ Moreover, it plays a central role in DNA recombination^[36]^ and mitotic chromosome segregation.^[37]^ Recent studies found that elevated SMC3 expression was related to poor outcome in colon cancer. SMC4, located in 3q25.33, was found highly expressed in HCC patients.^[11]^ In addition, high expression level of SMC4 could be an independent factor to predict poor survival in colorectal cancer,^[13,38]^ and is also related to the aggressiveness of glioma.^[39]^ In the present study, it was found that the expression of SMC2, SMC3 and SMC4 in HCC tissues were significantly higher than in normal liver tissues. Via univariate and multivariate Cox proportional hazards regression analyses, the prognostic value of SMC2, SCM3, and SMC4 in HCC was affirmed and high expression of SMC2, SMC3, and SMC4 were associated with poor PFS.

The SMC5/6 complex, which is essential for eukaryotes, is currently the least known of SMC complexes. SMC5/6 plays a key role in DNA repair due to promoting the separation of recombinant intermediates. In addition, SMC5/6 may impact cell proliferation in the G2 phase of mitosis, thereby affecting cancer progression, but its function in the development of HCC remains unknown.^[40]^ In the present study, no difference was found in the expression of SMC5 between HCC and normal tissues. However, SMC6 was highly expressed in HCC tissues, which was related to poor PFS in HCC patients.

By using updated public resources, we accessed the expression and prognosis of SMC family members in patients with HCC. What’s important, the high mRNA expression of SMC2, SMC3, and SMC4 were found to be associated with worse PFS, these findings suggested that individual SMC family members, especially SMC2, SMC3, and SMC4, are potential relative factors of prognosis in HCC and forecast that the use of SMC inhibitor targeting SMC2, SMC3, and SMC4 may be a practical strategy for HCC therapy.

Although bioinformatics analysis could explore the role of SMC family members in a short time and convenient manner, limitations of our study still need to be considered. Further studies with more datasets should be included to show more evidence of SMC members on the prognostic value in HCC.

## 5. Conclusion

This study discussed the gene expression profile of SMC family members in HCC and proved the biological and prognostic values of SMC family members in HCC. We found that increased expression of SMC2, SMC3, and SMC4 might play a significant role in HCC, and could be useful as molecular markers to identify high-risk patients, Our results also provide insights for further research of SMC family members as potential tumor therapeutic target in HCC.

## Author contributions

WY and QZ designed the research study and analyzed the data from public database. WY, DW, and HZ were involved in data analysis. WY was responsible for writing of manuscript. JH, LL and JZ contributed to the revised manuscript. All authors reviewed the manuscript.

**Conceptualization:** Dan-Dan Wang, Su-Jin Yang.

**Data curation:** Wei Yan.

**Formal analysis:** Jinny Huang, Qian Zhang.

**Investigation:** Wei Yan, Jun-Chen Hou.

**Project administration:** Ling Lu.

**Resources:** He-Da Zhang.

**Software:** Wei Yan, Su-Jin Yang, Jian Zhang, Qian Zhang.

**Writing – original draft:** Qian Zhang.

**Writing – review & editing:** Qian Zhang.
